# Chatbots as a Tool to Scale Mentoring Processes: Individually Supporting Self-Study in Higher Education

**DOI:** 10.3389/frai.2021.668220

**Published:** 2021-05-13

**Authors:** Alexander Tobias Neumann, Tamar Arndt, Laura Köbis, Roy Meissner, Anne Martin, Peter de Lange, Norbert Pengel, Ralf Klamma, Heinz-Werner Wollersheim

**Affiliations:** ^1^ Chair for Databases and Information Systems, RWTH Aachen University, Aachen, Germany; ^2^ Institute of Educational Sciences, Leipzig University, Leipzig, Germany

**Keywords:** automated feedback, chatbot, mentoring, self-study activities, technology-enhanced learning

## Abstract

Like most curricula in the humanities and social sciences, the curriculum of pre-service teacher training in educational sciences often includes time-consuming reading and writing tasks, which require high quality support and feedback in a timely manner. A well-known way to provide this support to students is one-to-one mentoring. However, limited time and resources in the German university context require to effectively scale the benefits of individual feedback. The use of scalable technologies to support learning processes seems to be promising, but its development usually requires a deep technical understanding. With an interdisciplinary approach, this contribution investigates how personal mentoring can be made available to as many students as possible, taking into account the didactic, organizational and technical frameworks at universities. We describe the development and implementation process of two chatbots that both aim to support students of educational sciences in their self-study of the seminar topics and literature. The chatbots were used by over 700 students during the course of 1 year and our evaluations show promising results that bear the potential to improve the availability of digital mentoring support for all students.

## 1 Introduction

Self-study activities are a central part of the learning process in higher education (Dörrenbächer and Perels, 2016). A well-known approach to support students in their learning process is one-to-one mentoring, which describes a dyadic relationship between a mentor with expert knowledge and a mentee (Nora and Crisp, 2007; Hattie and Yates, 2014). Traditionally, face-to-face mentoring requires high effort in one-to-one situations because of its holistic conversational processes and therefore does not scale easily for numerous students. Still, an active relationship and frequent interaction between the two parties are crucial parts for successful mentoring (Cornelius et al., 2016).

Chatbots are conversational interfaces that allow humans to interact with software using natural language, without space or time constraints. This makes them a promising means to scale mentoring in higher education that can provide individual learning support and feedback to more students than it would be possible with a small number of human mentors. It has been shown that chatbots used for mentoring provide the capability to encourage mentees, especially when learning factual knowledge (Mendez et al., 2019), sending them reminders of university activities and events, but also of lessons and deadlines (Dibitonto et al., 2018).

In this paper, we present both the design and scientific methods to scale mentoring processes through the development and utilization of technologies in the field of educational sciences. Our testbed is an educational science course within the curriculum of pre-service teacher training at a German university, attended by around 800 students each winter and summer term. The didactic design of the educational science course is heavily based on literature to be read in self-study. Course evaluations of past terms showed that students wished for more support in dealing with the numerous reading assignments in order to understand them and to be prepared for the exam (Pengel et al., 2020). In the winter term 2018/2019, an interdisciplinary team of educational- and computer-scientists started to develop ideas on how to better support students by combining the ideas of traditional mentoring and educational technology. The outcomes of this process ultimately lead to the results presented in this paper. We developed two chatbots, FeedBot and LitBot, that provide individual support for students’ self-study, specifically in dealing with seminar literature and recommending study material. With our approach, we suggest a way to provide automated feedback and recommendations to students where otherwise no individual feedback would be possible or significant additional staff would be required. It enhances traditional one-to-one mentoring with novel educational technologies and conversational Artificial Intelligence (AI). The interdisciplinary approach, a large-scale evaluation testbed and the conception, and also creation of bots by mentors with non-technical backgrounds provide a new perspective on creating technology enhanced mentoring support.

The remainder of this paper is structured as follows. We start by providing a background to our research in Section 2, before we describe the technical basis and architecture of our approach in Section 3. In Section 4 we present the development process of the FeedBot that offers writing tasks on seminar literature and provides automated feedback on the resulting students’ texts. We then continue with Section 5 by describing the development process of the LitBot, that individually supports students’ reading process. Our evaluations are presented in Section 6. Finally, Section 7 concludes this paper.

## 2 Background and Related Work

The first chatbots were created using rule-based approaches, which date back as far as to the 1960s, with ELIZA Weizenbaum (1966) being the first publicly demonstrated chatbot. These chatbots had a set of predefined rules, which parsed the input of a user and categorized it according to these rules, replying with mostly predefined statements. With the advancement of AI technology, in particular in the domain of Natural Language Understanding (NLU), chatbots and conversational agents in general have gained increased interest and adaption in both academia and industry. The majority of surveys on this subject categorize these works according to their used models, which are either retrieval-based or generative (Hussain et al., 2019; Agarwal and Wadhwa, 2020). Retrieval-based chatbots are trained to provide the most matching response from a database of predefined responses, according to the user input. The responses are based on existing information, and techniques like keyword matching and deep learning are used to identify them. One prominent example of a retrieval-based chatbot is ALICE, which uses the just as prominent Artificial Intelligence Markup Language (AIML) (Wallace, 2009). But retrieval-based chatbots are limited to only predefined responses and can not generate new output. Chatbots that use a generative model can generate new content based on conversational training data. Generative chatbots use techniques like (un-)supervised learning, reinforcement learning, and adversarial learning for their training. Another way of differentiating chatbots is by splitting them in task-oriented and non task-oriented. While the former ones are created to assist the user in solving a specific task, the latter have as goal to provide a conversational partner to the user, without having in mind a specific task that should be tackled during the conversation. In this contribution, we present two task-oriented chatbots that are based on a framework that uses retrieval-based assessment of user input.

## 3 Technical Basis and Architecture

To create the chatbots, we use the Social Bot Framework (SBF) (Neumann et al., 2019; Neumann et al., 2020), a Web-based collaborative modeling tool. Aiming to support collaborative bot development for users both with and without a technical background, the SBF offers a visualized model of the bot and allows for easy drag and drop interactions with elements like user intents and bot messages. For the intent recognition, the SBF integrates the open source conversational AI platform Rasa.^1^ To be able to recognize the intents expressed in a user message, a list of intents in accordance with the chatbot’s functional scope has to be created and for each intent, a number of exemplary intent utterances with optional marked entities needs to be added to train the NLU model. Thus, in addition to the model-driven creation of the chatbot, the SBF offers a Web-based collaborative text editor to specify and extend the list of intents known to the chatbot.

To generate and visualize automated feedback on writing tasks, our FeedBot utilizes the REST API of T-MITOCAR (Pirnay-Dummer and Ifenthaler, 2011), a software applying computational linguistics to analyze the knowledge structures of a text and visualize it in a graph representation. Additionally, for the utilization within our LitBot, we created an issue-specific knowledge graph (Meissner and Köbis, 2020), which builds upon Semantic Web technologies like RDF, triple stores and SPARQL. The actual knowledge graphs were created by manually extending the results of T-MITOCAR, which received an extract of the testbed’s course book as input data. In the following, this is referred to as annotated knowledge graphs.

**FIGURE 1:**
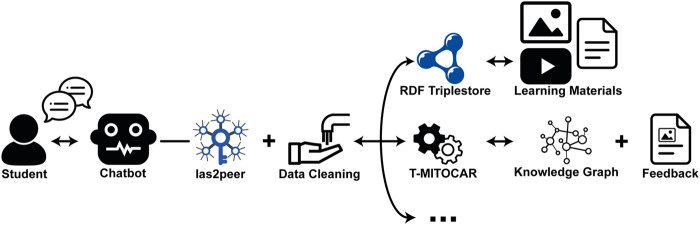
Deployed architecture: The student communicates with the las2peer based chatbot which can access different services like T-MITOCAR or (RDF) databases to provide mentoring support.

We deployed the SBF and the previously described tools in a kubernetes cluster as described in Klamma et al. (2020). Figure 1 provides a simplified view on the data flow of the system. Students interact with the respective chatbot via a messenger, in our case Rocket. Chat.^2^ The modeled chatbots rely on las2peer (Klamma et al., 2016), a peer-to-peer framework for distributed services, with which we ensure secure communication through strong asymmetric encryption. The data the chatbots receive is cleaned before it is further processed by mentoring tools, which then correspond again with the bot, utilizing the las2peer network. Finally, the chatbot is able to provide the results back to the students. In the following sections, we describe the functionality of the two bots in detail.

## 4 Writing Tasks and Feedback: FeedBot

FeedBot is a chatbot that supports students in their self-study, particularly in dealing with seminar literature. It does so primarily by offering writing tasks on the literature and providing automatically generated feedback in the form of graphs that represent an association network for the students’ texts. FeedBot also provides FAQ-style answers to students’ about the seminar and the course of study.

### 4.1 Background

In the context of mentoring in higher education, feedback is highlighted as an essential tool for making students’ skill and knowledge levels visible, thus helping students to monitor their own learning process (Hattie and Timperley, 2007; Hattie and Yates, 2014). One of our main goals is to provide individual feedback in a scalable way. T-MITOCAR uses computational linguistic analysis to automatically construct representations of knowledge from prose texts. In our case, the feedback is composed of the analysis of the student text and also a comparison of the knowledge structure from the student text with the corresponding knowledge models of the seminar literature.

### 4.2 Summer Term 2020

Our bot creation process started by collecting possible student intentions and corresponding example sentences with matching responses from the bot in a simple spreadsheet. In our initial version we had a set of rather generic messages like greetings, saying goodbye, referring to other contacts for help or reacting to expressions of gratitude or bad behavior, as well as scenario-specific messages such as showing current writing assignments, accepting submissions and transmitting the automatically generated feedback. This was extended by a set of Frequently Asked Questions (FAQ), that were based on a FAQ collection that had already been maintained over previous terms. For the summer term 2020 we added more FAQ regarding COVID-19 related restrictions and changes in the educational science course.

Once the anticipated content of the chatbot was created, we built the chatbot model using the SBF. The list of intents and example sentences described above were transformed into the appropriate intent-entity format for the Rasa integration using the markdown editor in the SBF in order to train the NLU model. While the initial version included all the desired features mentioned, internal tests showed that the set of FAQ was too large and the description of corresponding intents not yet mature enough to be included in the Rasa framework and the chatbot. Therefore, we decided to focus on the functions concerning writing tasks and providing automatically generated feedback for the first version of FeedBot and include only a small number of FAQ. The feedback was initially provided directly within the chat as a graphical representation of the knowledge model of the student’s text, but this was revised from a didactic point of view, since a pure presentation of a graph cannot easily be interpreted by students. Therefore, our next step was to create a template for annotated knowledge graphs which was filled with the feedback graphs and short textual explanations to contextualize them. Before FeedBot was made available to students, it was tested internally by the lecturers of the educational science course. Finally, at the end of the summer term, the bot was made available to students, offering them learning support during exam preparation.

### 4.3 Winter Term 2020/2021

We made FeedBot continuously available throughout this term, while simultaneously enhancing it. In addition to adjustments from the findings of the previous term, we also made some changes based on didactic considerations in order to further integrate FeedBot into the course. Therefore we decided to give more guidance by providing the writing tasks in several staggered intervals throughout the winter term.

**FIGURE 2:**
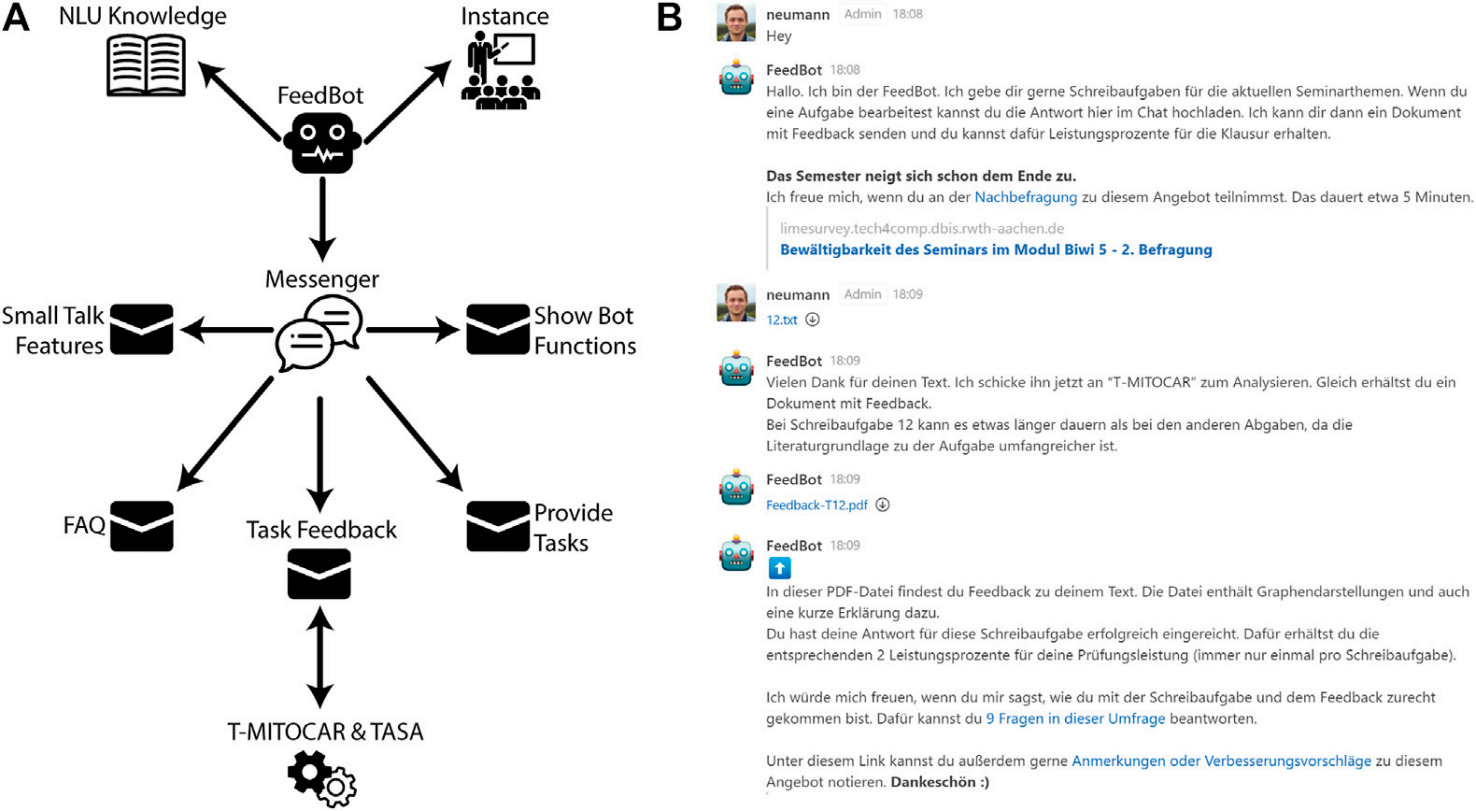
**(A)** Simplified model of the FeedBot. Messages grouped by functional scope. **(B)** Rocket.Chat conversation with FeedBot (greeting, sending task submission and receiving feedback).

Figure 2A shows the resulting bot model after the described adjustments were made. With the modeled functions the bot can respond to small talk, provide a list of its available functionalities, give answers to a small set of FAQ, provide writing tasks and give feedback on finished tasks. Figure 2B shows an excerpt of a conversation with FeedBot. After a welcoming message, FeedBot refers to a user survey. The student then sends a completed writing task to the bot. FeedBot informs the student about how his text is being processed. After a few seconds, FeedBot transmits the feedback generated with T-MITOCAR together with a brief explanation including a link to a survey on user satisfaction and user feedback.

## 5 Reading Support: LitBot

As described in Section 1, our educational science course testbed is heavily based on literature the students ought to read. Our LitBot aims to support and mentor students with their reading.

### 5.1 Background

With LitBot we want to accompany the reading with exercises and thereby motivate and structure self-study. When reading factual texts, readers must draw on their prior knowledge. Prior and domain specific knowledge affects and contributes to the understanding of a text and learning from a text (Kintsch, 1988; McNamara and Kintsch, 1996). Activating prior knowledge before reading can help identify relevant information in the text and enable connected learning (Kalyuga, 2005). Therefore, we designed LitBot to provide exercises to activate prior knowledge before a student has read the text (Rq 1). Additionally, after reading, LitBot provides prompts to reflect on the text (Rq 2) and recommendations for further material based on the students’ specific interests regarding the reading material (Rq 3).

### 5.2 Winter Term 2020/2021

**FIGURE 3:**
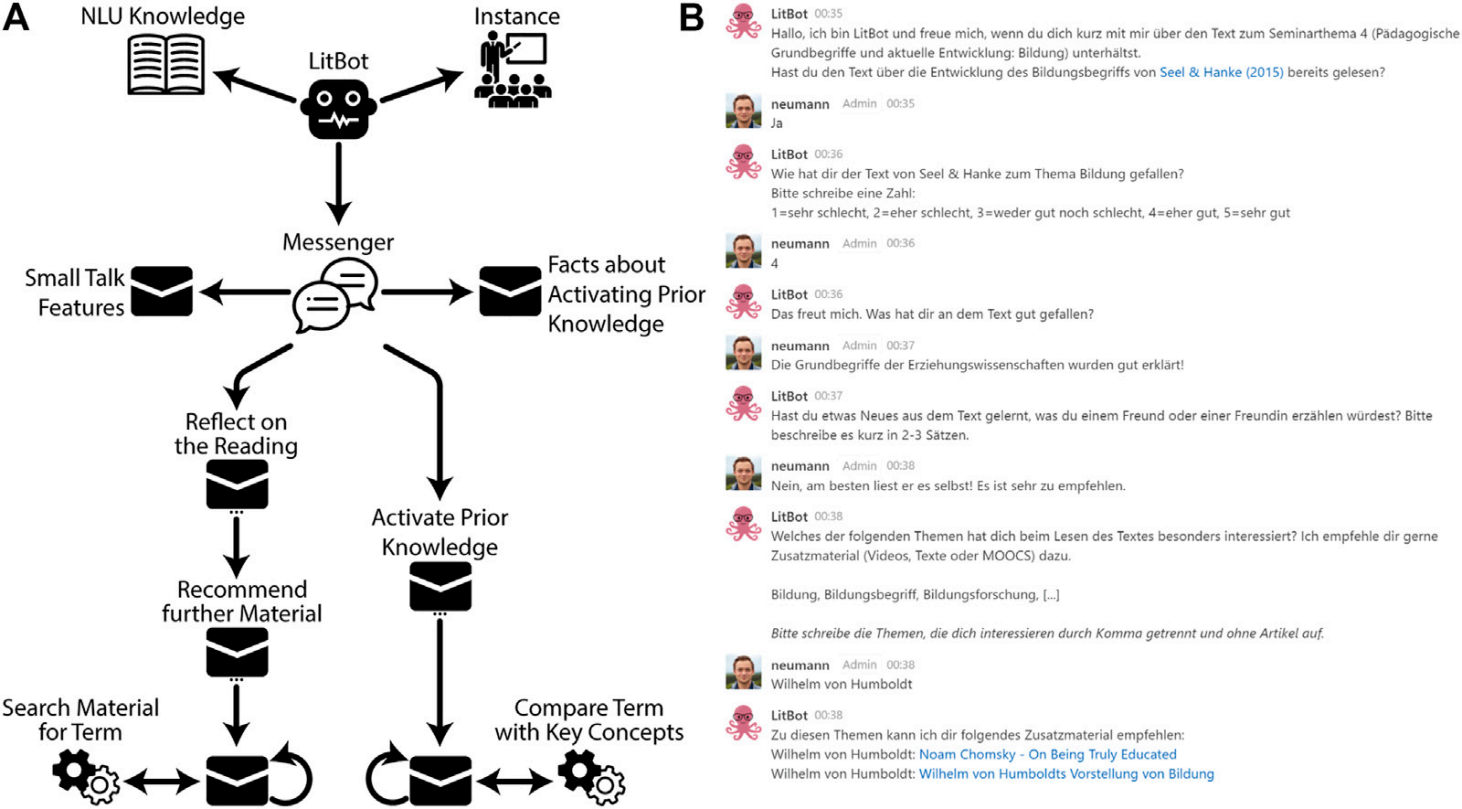
**(A)** Simplified model of the LitBot. Messages grouped by functional scope. **(B)** Rocket.Chat conversation with LitBot (greeting, getting material recommendations).

To implement the described features of LitBot, we mapped out a sequential chatbot conversation to guide students through the exercises before and after reading. The resulting (simplified) bot model (see Figure 3A) shows the tree-like structure with two sequential paths.

As mentioned in Section 4.2, LitBot utilizes issue-specific annotated knowledge graphs, which are rather complex and highly technical. The chatbot extracts the contained information from these and presents them in a more human-readable fashion, thereby acting as an easy-to-use interface between annotated knowledge graphs and students.

The chatbot and the annotated knowledge graphs were implemented during the winter term. For the initial field study and evaluation, we used one of the mandatory texts in our educational science course, which presents various definitions on the concept of education. This text was transferred to a graph by using the T-MITOCAR software. The graph was then annotated by lecturers in a semi-automated workflow, as outlined in Meissner and Köbis (2020).

Figure 3B shows an extract of a conversation with LitBot. The bot opens up the conversation with a greeting and the question, whether the student has already read the mandatory text about the concept of education (which, in this example, is answered positive). If the student would not have read the text yet, the chatbot would start with an exercise to activate the students’ prior knowledge concerning the content of the text (Rq 1), asking questions like “What are the first five words that come to your mind when you hear the word education?“. The association the student enters are then checked against the key concepts of the text in order to match them to the students’ expectations of the text. These key concepts are specified in the annotated knowledge graph. LitBot reports back which of the given terms correspond with the key concepts.

Coming back to the depicted example conversation, the chatbot prompts the student to reflect on the text (Rq 2) by asking questions, e.g., “How did you like the text?”; “Did you learn something new from the text that you would tell your friend?”. These questions not only encourage students to reflect on the reading, but the written responses can also be used by course instructors as feedback that helps them plan sessions and review course material.

The sample conversation depicted in Figure 3B ends with the LitBot recommending further learning material to the student (Rq 3). Subsequently, LitBot asks the student “What was especially interesting for you regarding the text that you have read?”. For this, the bot presents a list of concepts from the texts’ annotated knowledge graph for the student to choose from. The chatbot then resorts to the knowledge graph and searches within it for additional material like links to further reading, videos or movies.

## 6 Evaluation

We evaluated our approach in three steps. We started with an evaluation of the applicability of creating bots with the help of the SBF, within the context of educational science mentoring. Then, we undertook two (partly simultaneous) evaluations of both the FeedBot and the LitBot.

### 6.1 Workshop on Social Bot Creation

Our chatbots are conceptualized and created by lecturers of the educational science course. In order to kick-off the development processes, we conducted an introductory workshop on the SBF. The workshop took place online, with four sessions consisting of three to six participants. During the workshop, participants had to extend an exemplary chatbot model. At the end, the participants were asked to complete a questionnaire to examine the framework’s usability according to the System Usability Scale (SUS) (Brooke, 1996), using a Likert scale ranging from 1 to 5. Out of the 15 participants taking part in the workshop, 12 completed the survey. The results are visualized in Table 1. As one can see, results mostly supported the hypothesis, that the platform was well suited for its purpose of integrating the non-technical participants into the actual bot development process. Most participants showed real interest in using the framework, additionally expressing they found themselves now able to model a bot on their own or with little help. With this workshop providing initial positive reactions to the framework, we continued creating the Feed- and LitBot, whose evaluations we present in the upcoming two sections.

**TABLE 1:** Results of the usability questionnaire. Answers were provided in ordinal scale where 1 ≙ “strongly disagree” and 5 ≙ “strongly agree” (n = 12).

Question	Average	SD
I needed to learn a lot of things before I could get going with the social bot framework	2.33	± 1.11
I felt very confident using the social bot framework	2.83	± 0.79
I found the social bot framework very cumbersome to use	2.08	± 0.97
I would imagine that most people would learn to use the social bot framework very quickly	3.24	± 1.16
I thought there was too much inconsistency in the social bot framework	1.92	± 1
I found the various functions in the social bot framework were well integrated	4.08	± 1
I think that I would need the support of a technical person to be able to use the social bot framework	2.42	± 1
I thought the social bot framework was easy to use	3.25	± 1.16
I found the social bot framework unnecessarily complex	2.08	± 1.27
I think that I would like to use the social bot framework frequently	4.17	± 1.15

### 6.2 FeedBot

In the winter term we had 831 students registered in the educational science course. Over the course of this term, 715 students used the chatbot to submit over 8,400 text documents in total. Six students shared their impressions of FeedBot in a first qualitative survey during the term. For the evaluation of the open questions, a content analysis was conducted, which categorically clusters the students’ answers and thus systematizes significant elements with corresponding anchor examples, leading to inductive-deductive category formation. This serves the systematic processing of qualitative data, e.g., to avoid unsystematic picking out of individual quotes and examples. The answers repeatedly addressed interaction that play a significant role in the use of chatbots in higher education. Interaction with the chatbot was described to work well, but still the desire was expressed to be able to talk to a “real” person in case of ambiguities: “[…] It would be helpful to have a ‘Talk to staff member’-button or something, so that a real person could then intervene as support if necessary […]”. This corresponds to the need for communication in a technology-based learning environment with the problem of isolation due to the lack of personal exchange between the actors (Nebel, 2017). Initial insights into the use of the bot also showed that students rarely used the bot in an exploratory way, meaning that they did not try to discover the full range of functions the chatbot offered. In addition to these didactic and content-related aspects, additional remarks concerned the usability of the bot and problem handling, intent detection and latency issues. We will use this feedback for future iterations of the chatbot.

### 6.3 LitBot

LitBot was initially tested in a seminar session on the text about the concept of education, carried out online in December 2020. Students were asked to use the chatbot and evaluate it in a qualitative survey. The answers of the 13 students that participated in the qualitative survey are not representative, but gave us a first impression on how to improve and expand the LitBot. The overall perception was positive. The majority of students stated that the chatbot could motivate them to read a seminar text (n = 10) and support their self-study (n = 10). Six students would have liked an extension for more seminar texts and topics and nine students would recommend LitBot to fellow students.

The standardized and not personalized chatbot answers, that do not respond directly to students’ written answers, were criticized. To tackle this and to also avoid unnecessary repetition of questions asked by the chatbot, we plan to mark conversations which have already been passed through by the users as done. In addition, students stated in the evaluation that they wished for more reflection exercises to enable a more detailed work with the text. In the future, more text-related reflection and content exercises to guide the students’ reading are imaginable. For example, we would like to incorporate multiple choice questions about the reading, as well as definitions and further explanations to specific concepts of the reading. Regarding the question “Would you like a chatbot extension for more seminar topics?”, one student replied: “I don’t think that the bot is a replacement for personal discussions and interactions”. We totally agree with this statement and want to emphasize the aim of additional support for self-study purposes for students.

### 6.4 Limitations

The qualitative results are currently still quite modest. In the future, we plan to collect qualitative data within the chat conversation as well and not only from external survey platforms. Regarding the large amount of quantitative data we collected in the winter term, we are currently still evaluating this data and will report back on it at a later stage.

Additionally, we have to state that for each completed writing task of the FeedBot, we rewarded students with a small amount of credit for the exam. We acknowledge the fact, that this certainly influenced the usage frequency, but we also want to mention that the granted credit was only associated with the completion of the task, and not based on its quality.

## 7 Conclusion

In this contribution, we presented the development processes and evaluation of two chatbots. We showed an integration of different mentoring tools to support students’ self-study activities through the use of chatbots. The technical and didactic development, as well as the evaluation took place in parallel. The initial evaluations show promising results and identify aspects which require focus and improvement in future work.

Our repertoire of mentoring support tools is constantly growing. The presented bots will be further developed in an agile manner. We intend to use the large amount of data generated in the winter term 2020/2021 and future data from the chatbot to train and develop generative machine learning models. Also, we would like to be able to evaluate how students have used the chatbot and are currently pursuing path analysis approaches. Current trends show that adaptive learning is one of the key concepts for personalized learning in higher education (Alamri et al., 2021). Thus, we hope that in the future other courses in higher education can be enriched with the use of mentoring bots.

## Data Availability

The original contributions presented in the study are included in the article/Supplementary Material, further inquiries can be directed to the corresponding author.

## Footnotes

1
https://rasa.com/

2
https://rocket.chat/
